# Evaluation of Chat Generative Pre-trained Transformer and Microsoft Copilot Performance on the American Society of Surgery of the Hand Self-Assessment Examinations

**DOI:** 10.1016/j.jhsg.2024.10.001

**Published:** 2024-11-13

**Authors:** Taylor R. Rakauskas, Antonio Da Costa, Camberly Moriconi, Gurnoor Gill, Jeffrey W. Kwong, Nicolas Lee

**Affiliations:** ∗ College of Medicine, Florida Atlantic University, Boca Raton, FL; † Department of Orthopaedic Surgery, University of California San Francisco, San Francisco, CA

**Keywords:** AI, LLM, ChatGPT, Education, Examination, Certification

## Abstract

**Purpose:**

Artificial intelligence advancements have the potential to transform medical education and patient care. The increasing popularity of large language models has raised important questions regarding their accuracy and agreement with human users. The purpose of this study was to evaluate the performance of Chat Generative Pre-Trained Transformer (ChatGPT), versions 3.5 and 4, as well as Microsoft Copilot, which is powered by ChatGPT-4, on self-assessment examination questions for hand surgery and compare results between versions.

**Methods:**

Input included 1,000 questions across 5 years (2015–2019) of self-assessment examinations provided by the American Society of Surgery of the Hand. The primary outcomes included correctness, the percentage concordance relative to other users, and whether an additional prompt was required. Secondary outcomes included accuracy according to question type and difficulty.

**Results:**

All question formats including image-based questions were used for the analysis. ChatGPT-3.5 correctly answered 51.6% and ChatGPT-4 correctly answered 63.4%, which was a statistically significant difference. Microsoft Copilot correctly answered 59.9% and outperformed ChatGPT-3.5 but scored significantly lower than ChatGPT-4. However, ChatGPT-3.5 sided with an average of 72.2% users when correct and 62.1% when incorrect, compared to an average of 67.0% and 53.2% users, respectively, for ChatGPT-4. Microsoft Copilot sided with an average of 79.7% users when correct and 52.1% when incorrect. The highest scoring subject was *Miscellaneous*, and the lowest scoring subject was *Neuromuscular* in all versions.

**Conclusions:**

In this study, ChatGPT-4 and Microsoft Copilot perform better on the hand surgery subspecialty examinations than did ChatGPT-3.5. Microsoft Copilot was more accurate than ChatGPT3.5 but less accurate than ChatGPT4. The ChatGPT-4 and Microsoft Copilot were able to “pass” the 2015–2019 American Society for Surgery of the Hand self-assessment examinations.

**Clinical Relevance:**

While holding promise within medical education, caution should be used with large language models as more detailed evaluation of consistency is needed. Future studies should explore how these models perform across multiple trials and contexts to truly assess their reliability.

In recent years, the rapid development of artificial intelligence (AI) has transformed a wide range of tasks and industries. Chat Generative Pre-Trained Transformer (ChatGPT) is an example of an AI model known as a large language model (LLM) that is trained on large amounts of internet data to complete users’ prompts in a conversational manner. Since its release in 2022 by OpenAI, it has gained significant popularity.^1^ The use of ChatGPT and other LLMs in the medical field has been explored increasingly as a potential tool for medical education and clinical decision making, although clinical applications have remained limited.^2^, ^3^, ^4^ The AI systems, such as ChatGPT, have the potential to revolutionize a variety of fields and hold significant promise. However, one of its current limitations is its accuracy and consistency.

One way to assess ChatGPT’s accuracy is to compare its performance directly to that of human test takers on examination questions. Examination performance of ChatGPT has been evaluated with a variety of tests. One study found ChatGPT demonstrated a proficiency comparable to a third-year medical student on the United States Medical Licensing Examination step 1 and step 2 examinations.^5^ Similar results have been found for medical licensing examinations across the globe.^6^^,^^7^ At an even higher level of training, ChatGPT was found to perform at the level of a first-year resident on the Plastic Surgery In-Service Examination and the Orthopaedic In-Training Examination.^8^, ^9^, ^10^ Advancing to subspecialties, Han et al^11^ found that ChatGPT-3.5 did not perform as well on hand surgery self-assessment questions from the American Society of Surgery of the Hand (ASSH) and did not receive a passing score on the 2004–2013 examinations. Similar findings were reported for ChatGPT-3.5 on the European Board of Hand Surgery diploma examination.^12^ However, a more recent study found that an updated version of ChatGPT, ChatGPT-4, scored proficient on the 2019 self-assessment, although still below the average physician.^13^ Bing’s Microsoft Copilot, an AI-powered interface built on the backbone of ChatGPT-4, has yet to be evaluated using medical examinations to our knowledge.

The purpose of this study is to determine the accuracy of ChatGPT-3.5 and the more advanced ChatGPT-4 and Microsoft Copilot on the ASSH 2015–2019 self-assessment examinations. We aim to analyze the ability of LLMs, such as ChatGPT, to answer complex medical questions within the subspecialty of hand surgery and explore their potential for educational use among hand surgery residents.

## Materials and Methods

No institutional review board approval was obtained given that the review did not involve experimentation of human or animal subjects, and the data reviewed are public. This observational study included 5 ASSH self-assessment examinations from 2015–2019. Each examination consisted of 200 questions, totaling 1,000 questions available for input. Each question was entered in a new chat window to prevent drawing information from previous chats. Data collection was performed in January 2024 by three investigators using a predetermined spreadsheet.

Questions were entered into the chatbot exactly how they would appear in the examination without any prompt to avoid impact on the output. When using this zero-shot prompt methodology, we anticipated the chatbot might refuse to provide a direct answer for some questions. For example, the response might summarize the question, provide instructive details on each answer choice, and conclude by saying, “It’s crucial to consult with a medical professional for accurate and up-to-date information.” When this occurred, the lack of definitive answer choice was recorded and a second trial was performed in a new chat window with the prompt, “Select the best answer choice for the following question:...”.

Publicly available models of ChatGPT, 3.5 and 4, were used to allow comparison between the two. An alternative platform, the Microsoft Bing Copilot, was used to allow comparison across different chat bots drawing from similar data. Primary outcomes included if an answer was provided without prompting, whether the answer was correct, if an answer was provided with the prompt when needed, and whether that answer was correct. The percentage of users who chose ChatGPT’s answer was recorded based on existing performance statistics available through ASSH, as well as the percentage that got the question correct. Each question was placed into one of the following categories: *Basic Science*, *Bone and Joint*, *Neuromuscular*, *Skin*, *Vascular*, *Ancillary*, *Miscellaneous*, and *Not Applicable* (2018 only). To assess accuracy across difficulty levels, complexity was determined by the percent of users who answered correctly, assuming that this percentage correlated with difficulty. Questions were grouped into four levels of difficulty, with one being the easiest and four being the hardest: (1 = 75%–100%, 2 = 50%–74.9%, 3 = 25%–49.9%, 4 = 0%–24.9%).

In the course of data collection, we encountered questions that incorporated images and videos. Although images cannot be inputted into ChatGPT-3.5, we decided to include questions with images and videos, aligning with our objective to assess accurately ChatGPT’s utility in a real-world educational context. The ChatGPT-4 did have the capacity to interpret images/figures, and these were entered into the prompt. For Microsoft Copilot, only one image could be entered at time. Consequently, when multiple images were present within a question, the images were compiled into a single document so that the chatbot had all available information.

Accuracy for each year, question type, difficulty level, and overall accuracy were recorded, and 95% confidence intervals were calculated for the overall accuracy. For correct and incorrect responses, concordance of the LLM with the majority of test takers also was determined. Student t-tests were conducted to compare performance among various LLM platforms as well as between LLMs and the typical ASSH user. We aimed for a power of 0.80 to detect a significant difference between the versions of ChatGPT-3.5 and ChatGPT-4 with an α of 0.05. To calculate the sample size needed to detect a significant difference, ChatGPT-3.5 was assumed to answer 58% of questions correctly, ChatGPT-4 was assumed to answer 68.9% of questions correctly.^15^ A sample size of 101 was required, making the 1,000 questions used sufficient.

## Results

ChatGPT-3.5 correctly answered 51.6% and ChatGPT-4 correctly answered 64.3%, demonstrating a statistically significant improvement (difference 12.7%, *P*<.0001). Microsoft Copilot also performed significantly better than ChatGPT-3.5 by 8.3% for a score of 59.9% (*P*=.0002), but worse than ChatGPT-4 (*P*=.0043). Of the 355 questions with images/videos, ChatGPT-3.5 answered 187 correctly, ChatGPT-4 answered 214 correctly, and Microsoft Copilot answered 225 correctly. All LLMs had the highest accuracy with the second difficulty level (50-74.9% of users answered correctly) and the lowest accuracy with the hardest difficulty level (0%–24.9% of users answered correctly).

### ChatGPT-3.5

The ChaGPT-3.5 performed with a total accuracy of 51.6% [48.5, 54.7] (Table 1*)*. This would translate to a passing score (50% except for general surgeons who require a 75% correct threshold to pass the examination) only part of the time, and scores were consistently lower than ASSH user averages: 59%, 65%, 66%, 72%, and 71% over the 5 years respectively (*P*=.001).

**Table 1:** Breakdown of Question Accuracy, With and Without Prompting, for ChatGPT-3.5, ChatGPT-4, and Microsoft Copilot

Question set	2015	2016	2017	2018	2019	Total
Total questions	200	200	200	200	200	1000
Text-only questions	142	136	124	123	120	645
Questions answered without prompt by ChatGPT3.5	170 (85%)	180 (90%)	190 (95%)	196 (98%)	194 (97%)	930 (93%)
Correct	93	105	103	105	93	499
Additional questions answered with prompt by ChatGPT3.5	24 (12%)	11 (5.5%)	8 (4%)	3 (1.5%)	2 (1%)	48 (4.8%)
Correct	11	5	1	0	0	17
Questions not answered by ChatGPT3.5	6	9	2	1	4	22
Total correct	104 (52%)	110 (55%)	104 (52%)	105 (52.5%)	93 (46.5%)	516 (51.6%)
Questions answered without prompt by ChatGPT4.0	183 (91.5%)	181 (90.5%)	186 (93%)	196 (98.0%)	193 (96.5%)	939 (93.9%)
Correct	120	115	117	128	141	621
Additional questions answered with prompt by ChatGPT4.0	16 (8.0%)	18 (9.0%)	8 (4.0%)	4 (2.0%)	2 (1.0%)	48 (4.8%)
Correct	7	9	3	3	0	22
Questions not answered by ChatGPT4.0	1	1	6	0	5	13
Total correct	127 (63.5%)	124 (62.0%)	120 (60.0%)	131 (65.5%)	141 (70.5%)	643 (64.3%)
Questions answered without prompt by Microsoft Copilot	158 (79.0%)	156 (78.0%)	158 (79.0%)	165 (82.5%)	177 (88.5%)	814 (81.4%)
Correct	104	119	111	123	102	559
Additional questions answered with prompt by Microsoft Copilot	20 (10%)	15 (7.5%)	14 (7.0%)	11 (5.5%)	11 (5.5%)	71 (7.1%)
Correct	16	5	10	6	3	40
Questions not answered by Microsoft Copilot	22	29	28	24	12	115
Total correct	120 (60%)	124 (62%)	121 (60.5%)	129 (64.5%)	105 (52.5%)	599 (59.9%)

**Figure 1:**
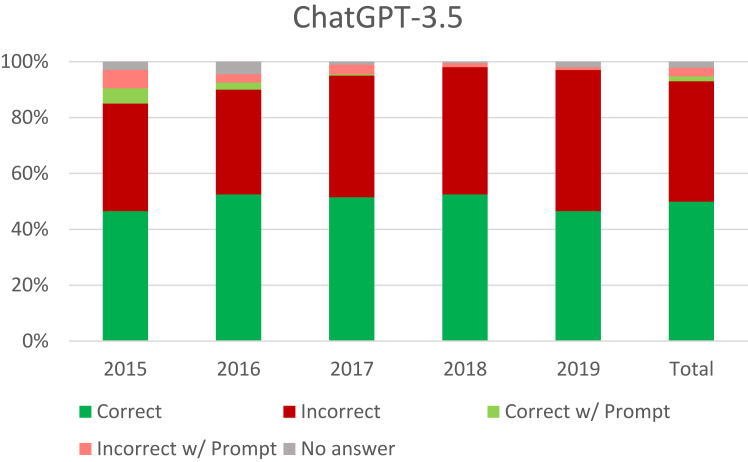
ChatGPT-3.5 performance.

The ChatGPT-3.5 answered 93% of questions without prompting, 4.8% with prompting, and failed to answer 22 (2.2%) with prompting (Fig. 1). When correct, the average number of users who also chose the right answer was 72.2%. When incorrect, users chose the correct answer 62.1% of the time. Of the 527 questions with the lowest difficulty (75%–100% correct), 256 were correct (48.6%; Table 2). Excluding *Not Applicable* because of small sample size, the top scoring question categories were *Miscellaneous* (60.0%), *Vascular* (59.7%), and *Basic Science* (58.8%). The lowest scoring categories were *Neuromuscular* (45.7%) and *Bone and Joint* (46.9%; Table 3).

**Table 2:** Accuracy Across Question Types: 3.5 = ChatGPT-3.5, 4 = ChatGPT-4, MC = Microsoft Copilot

Difficulty	Total	Number Correct (3.5)	Percent Correct (3.5)	Number Correct (4)	Percent correct (4)	Number Correct (MC)	Percent correct (MC)
1 (75%–100%)	527	256	48.6%	330	62.6%	285	54.1%
2 (50%–74.9%)	235	177	75.3%	206	87.7%	197	83.8%
3 (25%–49.9%)	179	68	38.0%	87	48.6%	91	50.8%
4 (0%–24.9%)	59	15	25.4%	20	33.9%	26	44.1%

**Table 3:** Accuracy Across Question Types

Question Type	Total	Number Correct (3.5)	Percent Correct (3.5)	Number Correct (4)	Percent Correct (4)	Number Correct (MC)	Percent Correct (MC)
*Basic Science*	153	90	58.8%	107	69.9%	99	65.4%
*Bone and Joint*	239	112	46.9%	139	58.2%	125	52.3%
*Neuromuscular*	208	95	45.7%	122	58.7%	112	54.3%
*Skin*	132	65	49.2%	82	62.1%	88	67.4%
*Vascular*	72	43	59.7%	50	69.4%	48	68.1%
*Ancillary*	83	43	51.8%	64	77.1%	50	60.2%
*Miscellaneous*	105	63	60.0%	75	71.4%	72	69.5%
*Not Applicable*	8	5	62.5%	4	50.0%	5	62.5%

MC, Microsoft Copilot; Difficulty 1, 75%–100% of users answered correctly; Difficulty 2, 50%–74.9% of users answered correctly; Difficulty 3, 25%–49.9% of users answered correctly; Difficulty 4, 0%–24.5% of users answered correctly.

### ChatGPT-4

ChatGPT-4 performed with a total accuracy of 64.3% [61.3, 67.3] (Table 1). This would translate to a passing score for trained hand surgeons and was more consistent with user averages (*P*=.5).

**Figure 2:**
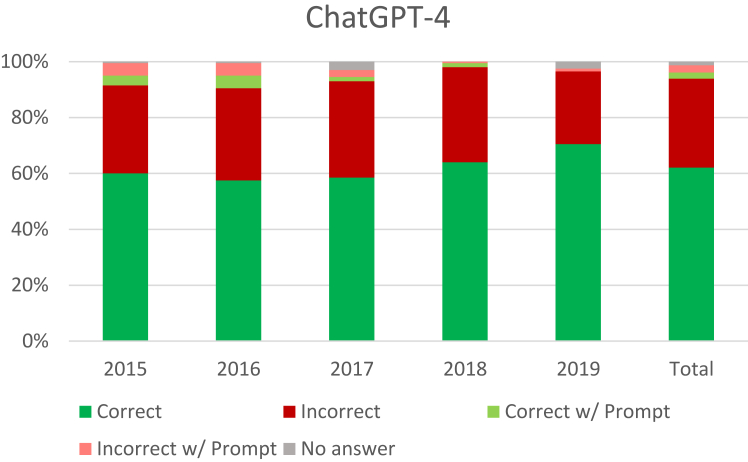
ChatGPT-4 performance.

The ChatGPT-4 answered 94.3% of questions without prompting, 4.8% with prompting, and failed to answer 13 (1.3%) questions with prompting (Fig. 2). When correct, the average number of users who also chose the right answer was 67.0%. When incorrect, users chose the correct answer 53.2% of the time. Of the 527 questions with the lowest difficulty (75%–100% correct), 330 were correct (62.6%; Table 2). Excluding *Not Applicable* because of small sample size, the top scoring question categories were *Ancillary* (77.1%), *Miscellaneous* (71.4%), and *Basic Science* (69.9%). The worst categories were *Bone and Joint* (58.2%) and *Neuromuscular* (58.7%) (Table 3).

### Microsoft Copilot

Microsoft Copilot performed with a total accuracy of 59.9% [56.0, 64.8] (Table 1). This would translate to a passing score but was not consistent with user averages (*P*=.03).

**Figure 3:**
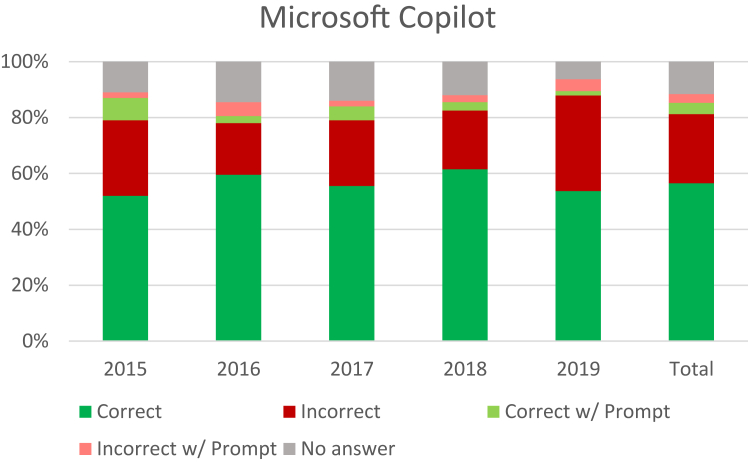
Microsoft Copilot performance.

Microsoft Copilot answered 81.4% of the questions without prompting, 7.1% with prompting, and failed to answer 115 with prompting (11.5%) (Fig. 3). When correct, the average number of users who also chose the right answer was 72.7%. When incorrect, the average who chose the correct answer was 59.1%. Of the 527 questions with the lowest difficulty (75%–100% correct), 285 were correct (54.1%) (Table 2). The top scoring question categories were *Miscellaneous* (69.5%), *Vascular* (68.1%), and *Skin* (67.4%). The worst categories were *Bone and Joint* (52.3%) and *Neuromuscular* (54.3%) (Table 3*)*.

## Discussion

The primary aim of this study was to assess ChatGPT versions 3.5 and 4, as well as Microsoft Copilot, on the ASSH self-examinations from 2015–2019. A key novelty of this work is the inclusion of Microsoft Copilot, which has not been evaluated previously in this context. Additionally, the inclusion of image-based questions adds a unique dimension. By analyzing this specific 5-year period, we provide a temporal comparison that offers new insights into how LLMs, like ChatGPT and Microsoft Copilot, perform on assessments over time. By examining this earlier time frame, we not only validate prior findings but also contribute additional layers of data that further solidify the relevance of LLMs in subspecialty education. We report the performance on 1,000 hand surgery questions, comparing scores against physicians sitting for competency benchmarking and between ChatGPT versions 3.5, 4, and Microsoft Copilot. While ChatGPT and similar LLMs must meet a higher standard for reliable use in medical education and examination preparation, our results demonstrate the rapid improvement of successive LLM generations and their growing proficiency in specialized levels of training.

A similar study by Han et al^11^ found ChatGPT’s performances on the 2004–2013 self-assessment examinations were poor, with the greatest accuracy being 42% (44% without image-based questions) for the 2012 form. With this result, the chatbot would not have achieved a continuing medical education credit. Although the chatbot showed promising results on the Orthopaedic In-Training Examination, hand surgery was a weak area with only 36.8% accuracy.^9^ The Han et al^11^ study included questions with images for ChatGPT-3.5, despite having no image interpretation program. Notably, ChatGPT-3.5 often referenced the image description while also revealing its lack of image interpretation in other responses. Future LLMs are likely to integrate image and figure analysis. Other OpenAI platforms, like Contrastive Language-Image Pre-training, have been developed, trained by matching images with descriptive captions.^14^

Our study demonstrates improved accuracy with ChatGPT 4 and Microsoft Copilot, built on ChatGPT-4’s foundational LLM. This may be because of the use of more recent self-assessments than in similar studies (2015–2019 vs 2004–2013). Arango et al^15^ found ChatGPT-3.5 and 4 score consistently above 50% on more current 2021 and 2022 self-assessments, supporting our findings. In that study, however, media-based questions were excluded. As surgery evolves and new data become available, what may have been correct 10 years ago may not be currently. Current examination averages increased from 59%–70% on 2015–2019 forms, progressing to 78% and 81% on the 2021 and 2022 forms.^15^ The study on previous years reported that the LLM gave less explanation for its answers on the 2004–2006 forms compared to the 2013–2017 forms.^11^ This further supports that as data evolve over time, ChatGPT has more relevant data available to pull from.

Passby et al^16^ reported ChatGPT-3.5 scored 63.1% on the Dermatology Specialty Certification Examination, which impressively improved to 90.5% using ChatGPT-4. Our study similarly showed ChatGPT-4 outperforming ChatGPT-3.5, likely explained by a more diverse training data set, model architecture, and training regimen.^17^ When comparing ChatGPT-4 and Microsoft Copilot, the former scored better overall, although Microsoft Copilot didn’t answer more questions. This can possibly be explained by the “Precise” conversation style chosen, which emphasizes evidence-based responses. A previous study supports the superiority of Copilot over ChatGPT-3.5 on an otolaryngology evaluation, yet it is unclear how many questions each LLM left unanswered.^18^ When compared to ChatGPT-4, Microsoft Copilot left more questions unanswered but had greater accuracy with the answered questions, arguable exercising greater precision and honesty with its limits. Future studies are warranted to explore discrepancies among ChatGPT-4 (subscription) versus Microsoft Copilot (free) despite operating on the same LLM.

The highest scoring subject was *Miscellaneous* and the lowest scoring subject was *Neuromuscular* in all versions, consistent with a study involving the 2019 examination.^13^ One possible explanation for the lower scores in *Neuromuscular* and *Bone and Joint* is the broadness of these categories, making up the bulk of orthopedic practice and recertification examination questions, in addition to their integration of physical examination and imaging findings. The small sample size of *Miscellaneous* may not fully explain its high score. As for question difficulty, it is unclear why the LLMs perform better on the second easiest level of difficulty. Our findings may be attributed to several factors, including question ambiguity, simplistic context, and data distribution. Large language models are trained to answer complex inquiries, possibly leading them to allocate less attention to relatively easy questions that lack sufficient phrasing.

The success of ChatGPT on specialized certification examinations demonstrates its potential usefulness for medical education and examination creation. Siu et al^15^ studied a variety of LLMs and how they approached a surgical case study. Across platforms, the reliability of information provided was deemed suitable for first-year surgical residents. Other studies depict similar findings at the medical student level as well, claiming that ChatGPT exhibits minimal self-contradiction and directional explanations.^9^ The ability of ChatGPT to pass complex medical examinations demonstrates potential for generating similar questions. Cheung et al^19^ looked at ChatGPT medical examination questions against human-created ones showing that the two were comparable in quality. The chatbot notably took 1/10 the time of human reviewers, showcasing immense efficiency to be leveraged by examination creators.

This study has several limitations. The ChatGPT-3.5 lacks image processing capabilities and while ChatGPT-4 has some limited image interpretation abilities, both models are unable to process video content. Visual assessment is critical for evaluating surgical professions; therefore, our results cannot be fully generalized. There are inherent limitations to LLMs based on the inconsistent data that they are trained on. Because ChatGPT draws on data freely available on the internet, it is subject to any bias within these data. Previous studies also have found variability in ChatGPT answers across trials. When using this technology, we must remain wary of LLM’s consistency when applying AI to medical education or clinical decision making. Finally, during this study’s data collection, we found Microsoft Copilot took notably longer than ChatGPT to generate a response, at times more than 5 minutes. The response time did not appear to relate to question length, but additional research could be performed to explore this relationship.

This study establishes an updated benchmark for ChatGPT’s performance on surgical subspecialty examinations. The ChatGPT-4 can pass the ASSH self-assessment examinations and shows promise in medical education at a subspecialty level. While LLMs are improving rapidly, and it is important to track their progress longitudinally, the ability to access images and reliably answer higher order questions is lacking. Caution is needed when incorporating ChatGPT into learning and practice. Additional research should continue to assess ChatGPT and other LLMs’ test-taking and clinical reasoning across various fields and scenarios.

## Conflict of Interest

No benefits in any form have been received or will be received related directly to this article.
